# Supplementary figures and images for: A Feasibility Study towards the On-Line Quality Assessment of Pesto Sauce Production by NIR and Chemometrics
Source: Foods. 2023 Apr 18;12(8):1679. doi: 10.3390/foods12081679 (PMC10137520; doi:10.3390/foods12081679)

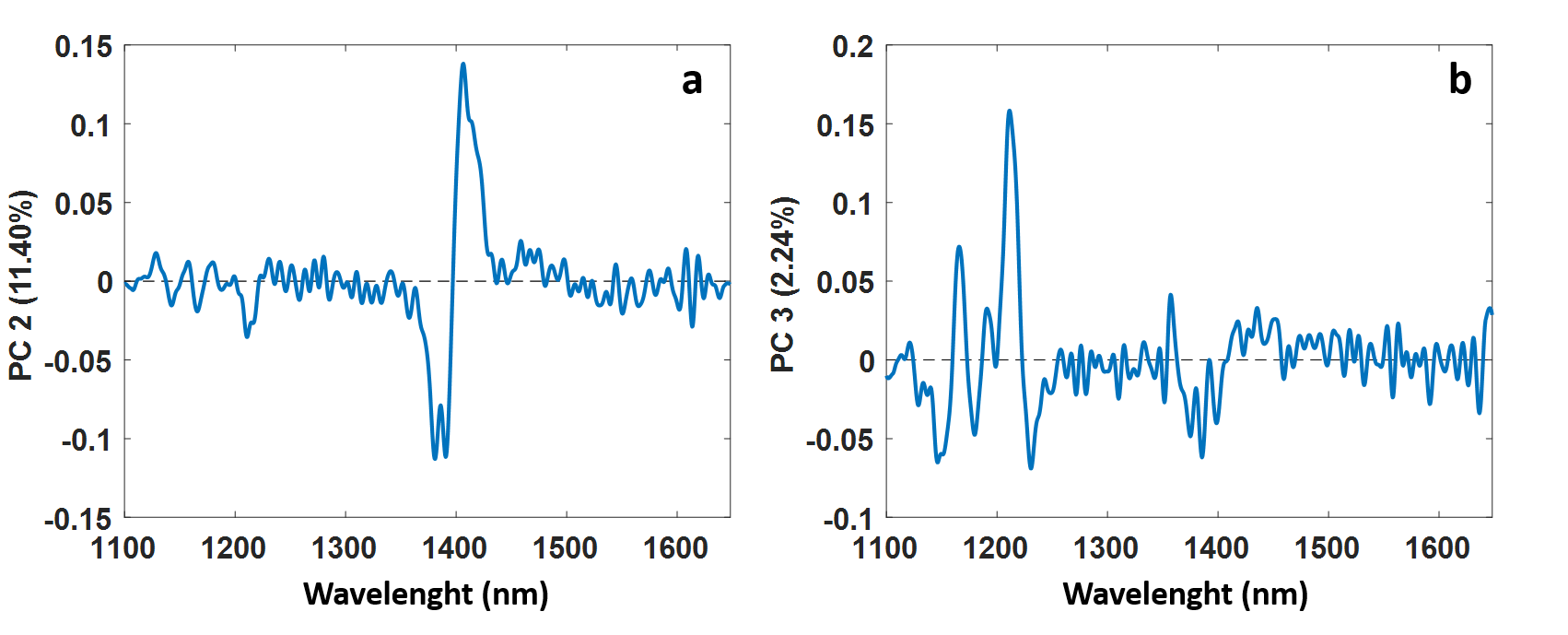

Supplement: Supplementary file 1 [file foods-12-01679-s001.zip › Figure. S1.tif]
